# Barriers to Digital Technology for Homebound Older Adults

**DOI:** 10.1093/geroni/igaf122.2793

**Published:** 2025-12-31

**Authors:** Sheryl Groden, MIchelle Sahli, Donna Case

**Affiliations:** University of Michigan-Flint, Flint, Michigan, United States; University of Michigan-Flint, Flint, Michigan, United States; University of Michigan-Flint, Flint, Michigan, United States

## Abstract

Many homebound older adults require digital literacy to use telehealth and online programs which have been shown to be helpful in decreasing social isolation. Our research addressed homebound older adults’ internet access, use of technology, and perceived barriers to digital technology. We partnered with our local Office on Aging, Meals on Wheels in two counties (Genesee and Washtenaw), and Homebound Services at the Genesee County Library. Key research questions included prevalence of internet access in homebound older adults, ways in which these older adults use technology, barriers to internet technology use, and how internet use would change if barriers were removed. We surveyed 61 homebound adults 60 years of age and older about their internet technology use and found that 24.6% did not have access to the internet. Respondents reported the following challenges to technology use: health or physical condition (44.3%), uncertainty on how to use devices (82%), unfamiliarity with options (68.9%), disinterest (42.6%), lack of device with internet access (18%), and discomfort in learning new things (50.8%). If these barriers were removed, 14.8% would start to use technology, 25.9% would use it more than they do now, 11.% still would not use technology and 27.9% technology use would not change. These findings provide evidence that internet access alone does not remove older adults’ barriers to digital literacy, and may help guide professionals when planning interventions and identifying the supports that would best facilitate elderly engagement with technology.

